# EUS-Guided Gallbladder Drainage Using a Lumen-Apposing Metal Stent for Acute Cholecystitis: Results of a Nationwide Study with Long-Term Follow-Up

**DOI:** 10.3390/diagnostics14040413

**Published:** 2024-02-13

**Authors:** Cecilia Binda, Andrea Anderloni, Edoardo Forti, Pietro Fusaroli, Raffaele Macchiarelli, Mauro Manno, Alessandro Fugazza, Alessandro Redaelli, Giovanni Aragona, Mauro Lovera, Thomas Togliani, Elia Armellini, Arnaldo Amato, Mario Luciano Brancaccio, Roberta Badas, Nicola Leone, Germana de Nucci, Benedetto Mangiavillano, Monica Sbrancia, Valeria Pollino, Andrea Lisotti, Marcello Maida, Emanuele Sinagra, Marco Ventimiglia, Alessandro Repici, Carlo Fabbri, Ilaria Tarantino

**Affiliations:** 1 Gastroenterology and Digestive Endoscopy Unit, Forlì-Cesena Hospitals, AUSL Romagna, 47121 Forlì-Cesena, Italy; monica.sbrancia@auslromagna.it (M.S.); carlo.fabbri@auslromagna.it (C.F.); 2 Gastroenterology and Digestive Endoscopy Unit, Fondazione I.R.C.C.S., Policlinico San Matteo Viale, 27100 Pavia, Italy; a.anderloni@smatteo.pv.it; 3 Digestive and Interventional Endoscopy Unit, Ospedale Ca’ Granda Niguarda, 20162 Milan, Italy; edoardo.forti@ospedaleniguarda.it; 4 Gastroenterology Unit, Hospital of Imola, University of Bologna, 40026 Imola, Italy; ptrfusa@gmail.com (P.F.); lisotti.andrea@gmail.com (A.L.); 5 Gastroenterology Unit, A.O.U.S. Policlinico S. Maria alle Scotte, 53100 Siena, Italy; r.macchiarelli@ao-siena.toscana.it; 6 Gastroenterology and Digestive Endoscopy Unit, Azienda USL Modena, 41121 Modena, Italy; m.manno@ausl.mo.it; 7 Division of Gastroenterology and Digestive Endoscopy, Department of Gastroenterology, IRCCS—Humanitas Research Hospital, 20089 Milan, Italy; alessandro.fugazza@humanitas.it (A.F.); alessandro.repici@hunimed.eu (A.R.); 8 Endoscopy Unit, San Gerardo Hospital, 20900 Monza, Italy; a.redaelli@asst-monza.it; 9 Gastroenterology and Hepatology Unit, Guglielmo da Saliceto Hospital, 29121 Piacenza, Italy; g.aragona@ausl.pc.it; 10 Digestive Endoscopy Unit, Fondazione Poliambulanza Istituto Ospedaliero, 25133 Brescia, Italy; mauro.lovera@poliambulanza.it; 11 Gastroenterology Unit, University Hospital Borgo Trento, 37126 Verona, Italy; thomas.togliani@gmail.com; 12 Digestive Endoscopy Unit, ASST Bergamo Est, 24060 Seriate, Italy; elia_armellini@hotmail.com; 13 Department of Gastroenterology, Valduce Hospital, 22100 Como, Italy; arnamato@gmail.com; 14 Division of Gastroenterology, S. Maria delle Croci Hospital, 48121 Ravenna, Italy; mario.brancaccio@auslromagna.it; 15 Digestive Endoscopy Unit, University Hospital, 09123 Cagliari, Italy; roberta.badas@hotmail.it; 16 Digestive Endoscopy Unit, Humanitas Gradenigo, 10153 Turin, Italy; nicola.leone@gradenigo.it; 17 Gastroenterology and Endoscopy Unit, ASST Rhodense, 20024 Garbagnate Milanese, Italy; germanadenucci1@gmail.com; 18 Gastrointestinal Endoscopy Unit, Humanitas-Mater Domini, 21100 Castellanza, Italy; benedetto.mangiavillano@mc.humanitas.it; 19 Digestive Endoscopy Unit, S. Michele Hospital, 09126 Cagliari, Italy; vallipolli@gmail.com; 20 Gastroenterology and Endoscopy Unit, S. Elia-Raimondi Hospital, 93100 Caltanissetta, Italy; marcello.maida@hotmail.it; 21 Department of Medicine and Surgery, School of Medicine and Surgery, University of Enna ‘Kore’, 94100 Enna, Italy; 22 Gastroenterology and Endoscopy Unit, Fondazione Istituto G. Giglio, Contrada Pietrapollastra Pisciotto, 90015 Cefalù, Italy; emanuelesinagra83@googlemail.com; 23 Directorate General of Medical Device and Pharmaceutical Service, Italian Ministry of Health, 00153 Rome, Italy; marco20miglia@gmail.com; 24 Department of Biomedical Sciences, Humanitas University, 20090 Milan, Italy; 25 Endoscopy Service, Department of Diagnostic and Therapeutic Services, IRCCS-ISMETT, 90100 Palermo, Italy; itarantino@ismett.edu

**Keywords:** acute cholecystitis, lumen apposing metal stent, EUS-guided drainage, EUS-guided gallbladder drainage

## Abstract

Background: Although endoscopic ultrasound-guided gallbladder drainage (EUS-GBD) using lumen-apposing metal stents (LAMS) has become one of the treatments of choice for acute cholecystitis (AC) in fragile patients, scant data are available on real-life settings and long-term outcomes. Methods: We performed a multicenter retrospective study including EUS-guided GBD using LAMS for AC in 19 Italian centers from June 2014 to July 2020. The primary outcomes were technical and clinical success, and the secondary outcomes were the rate of adverse events (AE) and long-term follow-up. Results: In total, 116 patients (48.3% female) were included, with a mean age of 82.7 ± 11 years. LAMS were placed, transgastric in 44.8% of cases, transduodenal in 53.3% and transjejunal in 1.7%, in patients with altered anatomy. Technical success was achieved in 94% and clinical success in 87.1% of cases. The mean follow-up was 309 days. AEs occurred in 12/116 pts (10.3%); 8/12 were intraprocedural, while 1 was classified as early (<15 days) and 3 as delayed (>15 days). According to the ASGE lexicon, two (16.7%) were mild, three (25%) were moderate, and seven (58.3%) were severe. No fatal AEs occurred. In subgroup analysis of 40 patients with a follow-up longer than one year, no recurrence of AC was observed. Conclusions: EUS-GBD had high technical and clinical success rates, despite the non-negligible rate of AEs, thus representing an effective treatment option for fragile patients.

## 1. Introduction

The management of acute cholecystitis (AC) has recently been revised and updated by the latest Tokyo Guidelines [1]. Although the gold standard of treatment is still represented by laparoscopic cholecystectomy regardless of the degree of severity, minimally-invasive approaches should be taken into consideration for “fragile patients”, that is, those affected by severe comorbidities and with poor performance status, considered high-risk or unfit for surgery [1].

**Figure 1:**
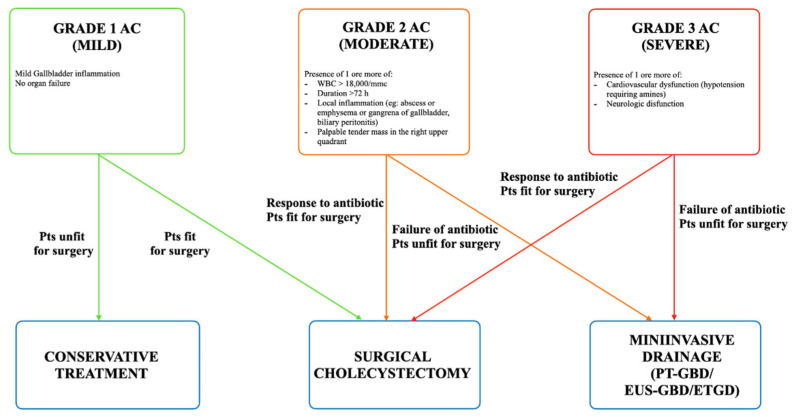
Severity grading of AC according to Tokyo guidelines.

Among nonsurgical treatments, endoscopic ultrasound gallbladder drainage (EUS-GBD), which was first described in 2007 [2], has demonstrated encouraging results and a good safety profile in the last decade [3,4,5,6,7,8,9]. Moreover, the introduction of lumen-apposing metal stents (LAMSs) in the endoscopist’s armamentarium has led to the widespread use of EUS-GBD, which nowadays represents an alternative to the well-assessed percutaneous gallbladder drainage (PT-GBD), overcoming the main limitations of catheter dislodgement and patient discomfort [10,11,12,13]. The updated Tokyo Guidelines for AC introduced EUS-GBD as a mininvasive option of treatment for patients affected by grade 2 and grade 3 AC, who are considered poor candidates for surgical cholecystectomy [1] (Figure 1).

However, data regarding the use of LAMSs in this cohort of patients are still sparse, mainly derived from a small series of patients and only one large multicenter retrospective study [5,7,9,14,15,16,17], mostly performed in tertiary referral centers. In addition, data are lacking about long-term follow-up, namely after more than 12 months, raising concerns about the risk of recurrent symptoms, as well as unexpected AEs.

The aim of this study is to evaluate the effectiveness and safety of EUS-GBD using LAMS for AC in a large cohort of patients at high risk for cholecystectomy, in real-life settings, involving centers with different volumes of procedures as well as different levels of endoscopist expertise.

## 2. Materials and Methods

In 2019, the Interventional Endoscopy and Ultrasound (i-EUS) group was formed in Italy as a result of a nationwide education initiative on the use of LAMS involving about 80% of centers that were “LAMS-users”. The main aim of the i-EUS group is to support and promote education initiatives in order to optimize the use of LAMS in clinical practice. The i-EUS group includes Centers all over Italy with different expertise in interventional endoscopy and LAMS use [18]. Expertise was defined in terms of the volume of EUS and ERCP per year, overall number of LAMS placed at the time of the study and type of indication for LAMS placement. Centers with less expertise were defined as those that performed fewer than 250 EUS/year, less than 200 ERCP/year and at the time of the study, had placed an overall number of LAMS < 20.

All the Centers of the i-EUS group who had the willingness to partcipate, were included in a multicenter retrospective data collection process of all procedures of EUS-guided drainage with LAMS for three major indications (pancreatic fluid collections (PFC), gallbladder, biliary), gathering data from 850 procedures. The study was approved by the institutional review board of each participating center (NCT03903523) and was performed in accordance with the Declaration of Helsinki.

The present retrospective multicenter study included 19 Italian secondary and tertiary endoscopy units that performed EUS-guided procedures using LAMS, including EUS-GBD. All patients undergoing EUS-GBD for AC and considered fragile or at high risk of cholecystectomy due to comorbities, namely patients with a Charlson Comorbity Index (CCI) ≥4, an American Society of Anesthesiologists physical status classification system (ASA-PS) score ≥3, and organ failure [1,19,20,21,22], were enrolled between June 2014 and July 2020. Inclusion criteria were: patients >18 years, ability to sign an informed consent form for the procedure, diagnosis of grade 2 and grade 3 AC, and EUS-GBD performed using LAMS. Exclusion criteria were: patients <18 years, pregnancy, and suspected or proven gangrenous or perforated gallbladder.

### 2.1. Outcomes

The primary outcomes were technical success, defined as correct deployment of LAMS, and clinical success, defined as improvement in clinical symptoms, namely right abdominal pain, nausea, vomiting, hypotension, and laboratory tests (including white blood cell count (WBC), c-reactive protein (CRP), total and direct bilirubin levels and transaminases).

The secondary outcomes were adverse event (AE) rate and severity, 30-day and overall mortality, and, not least, long-term outcomes of the procedure evaluating patients with survival and follow-up longer than 12 months.

### 2.2. EUS-Guided Gallbladder Drainage Procedure

The EUS procedures were performed using linear array echoendoscopes using carbon dioxide insufflation. Patients were under deep sedation or general anesthesia at the discretion of the anesthesiologist. A morphological assessment of the gallbladder was carried out under EUS-view, and based on the ultrasound results, the most suitable window of access—whether transgastric or transduodenal—was determined. After the identification of the puncture site, using color doppler, interposed vessels were excluded. When a cold system was used, the gallbladder was punctured with a 19-gauge needle, followed by aspiration of bile or contrast injection in order to confirm the correct positioning and placement of a 0.025- or 0.035-inch guidewire [16,23]. Over the guidewire, the needle track was dilated using a cystotome (settings: pure cut mode, 100 Watts) and/or a biliary balloon; then the delivery system of the stent was inserted, and the LAMS was deployed under fluoroscopic and endoscopic control.

If an electrocautery-enhanced LAMS (EC-LAMS) was used, the delivery system was inserted into the working channel and connected to the electrosurgical generator (settings: pure cut mode, 100 Watts). Access to the gallbladder was gained either using a previously placed guidewire or directly under EUS guidance using the “single-stage technique”. Once the delivery catheter was inside the gallbladder and the first flange was deployed under EUS guidance, the release of the second flange could be performed outside the working channel under direct endoscopic visualization or using the intrachannel technique [24].

**Figure 2:**
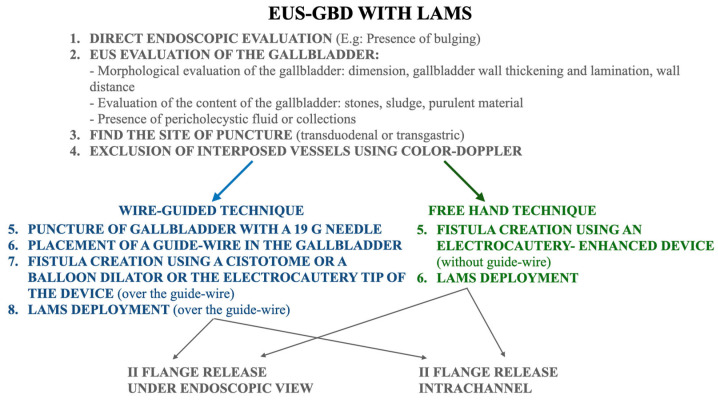
Procedural steps of EUS-GBD using LAMS.

Procedural steps are summarized in Figure 2.

### 2.3. Data

Data were compiled and extracted from a central online database. For each procedure, patient-related data on demographics, etiology of cholecystitis, presence of previous cholecystitis, medical therapy failure, use of warfarin or DOAC, gallbladder diameter, gallbladder features, concomitant percutaneous drainage, and previous endoscopic retrograde cholangiopancreatography (ERCP) with papillosphincterotomy for stones or transpapillary gallbladder drainage were collected. Procedural details, including type and size of LAMS used, deployment technique, site of approach, and procedural and deployment stent time were collected. Finally, post-procedural data, including length of hospitalization, other procedures performed, such as stent dilation, stone clearance, start of post-procedural enteral diet, gallbladder resection, were collected. AEs were graded according to the ASGE lexicon severity grading system [25], which is to date largely accepted and helps in standardizing severity and management. AEs were classified as immediate if they occurred during the procedure, early, when presenting within 14 days, and late, when presenting after 14 days from the EUS-GBD. Patients were followed up with periodic laboratory analyses and clinical visits at the discretion of the responsible endoscopist at each participating hospital.

### 2.4. Statistical Analysis

Continuous variables were reported as mean ± standard deviation, whereas categorical variables were summarized as frequency and percentage. Comparisons of variables were made by t-test and Chi square test as appropriate. Logistic regression models were performed to identify variables associated with the following outcomes: clinical success, 30-day mortality. Cox’s proportional hazard model was used to identify prognostic factors for overall mortality in a multiple regression analysis.

Considering the low number of events, to avoid the phenomenon of overfitting, the logistic regression models were built including a number of variables proportional to those of the observations. Variables considered in the models were selected through stepwise model selection and guided by clinical relevance.

For all analyses, a *p*-value less than 0.05 was considered statistically significant.

All statistical analyses were performed using SPSS v. 28.0 for Macintosh (SPSS Inc., Chicago, IL, USA).

## 3. Results

### 3.1. Study Population

A total of 116 patients (pts) (48.3% female) affected by AC were included. The mean age was 82.7 ± 11 years. In 15 pts (12.9%), AC was associated with malignancies (10/15) or complicated by empyema (3/15), Mirizzi’s syndrome (1/15) or cholecysto-duodenal fistula (1/15). Twenty-eight patients (24.3%) previously underwent ERCP for the concomitant presence of stones in the common bile duct (CBD), while in three cases, a previous endoscopic transpapillary gallbladder drainage (ETGBD) was attempted. Three patients (2.6%) had concomitant percutaneous drainage (PTGBD). The mean gallbladder major axis was 71.2 ± 35.8 mm, while the mean width was 59.4 ± 34 mm. More than half of the patients (63/116, 54.3%) had stones and sludge in the gallbladder, while in other cases, an acalculous (14/116, 12.1%) or purulent (39/116, 33.6%) gallbladder was found. Fourteen patients (12%) had a history of Warfarin or direct-acting oral anticoagulant (DOAC) intake, but in all cases, anticoagulants were suspended at least 4 days or more before EUS-GBD. Patients and their clinical characteristics are outlined in Table 1.

### 3.2. Gallbladder Drainage Procedure

In most cases (107/116, 92.2%), the Hot-Axios stent (Boston Scientific, Marlborough, MA, United States) was used, while the Nagi stent (Taewoong Medical, Gyeonggi-do, Seoul, Republic of Korea) was the stent of choice in the remaining patients (9/116, 7.8%). The most used LAMS was the 10 × 10 mm Hot-Axios stent (82/116, 70.7%) (Supplementary Table S1).

LAMS were placed transgastric in 44.8% (51/116) of patients, transduodenal in 53.3% (62/116) and transjejunal in 1.7% (2/116) due to the presence of altered anatomy. A single-stage access to the gallbladder was performed in 87.1% (101/116) of cases, while a guidewire was previously placed in 12.9% of patients (15/116). The release of the second flange was mainly performed intrachannel (76/116, 67.2%), and fluoroscopic control of the procedure was used in only 38.8% (45/116) patients. The mean procedure time (scope in to scope out) was 24.5 min.

### 3.3. Primary Outcomes

Technical success was achieved in 94% (109/116 pts) of cases, while clinical success was achieved in 87.1% (101/116 pts). All the biochemical examinations improved at two weeks after EUS-GBD drainage: WBC count moved from a mean of 16.13 (×10^3^/mm^3^) to a mean of 8.86 (×10^3^ /mm^3^), CRP from a mean of 21.21 mg/dL to a mean of 8.26 mg/dL, while total and direct bilirubin levels changed from 5.15 mg/dL and 4.14 mg/dl to 2.65 mg/dL and 1.96 mg/dL, respectively. The reduction was statistically significant for all the parameters (*p* < 0.05). The mean hospital stay was 11.2 ± 9 days, while the mean follow-up was 309 days.

### 3.4. Secondary Outcomes

AEs occurred in 12/116 pts (10.3%); 8/12 were intraprocedural, while 1 was classified as early (<15 days) and 3 as delayed (>15 days). According to the ASGE lexicon, two (16.7%) were mild, three (25%) were moderate, and seven (58.3%) were severe (Table 2). In three cases, complications, namely bleeding, AC recurrence and a cardiac arrest, were managed conservatively. In one case, intraprocedural bleeding from the gallbladder wall was managed endoscopically with adrenaline injection. In three patients, intraprocedural dislodgment of LAMS occurred, requiring urgent cholecystectomy in one patient, while an endoscopic approach was performed in the remaining two cases, namely defect closure of the gastric wall with an over-the-scope clip (OTSC) and placement of a second LAMS. Two cases of perforation and one of delayed LAMS migration required an urgent cholecystectomy with suturing of the duodenal wall. In one patient, LAMS was incorrectly placed in a renal cyst, with secondary development of sepsis and requiring LAMS removal. Lastly, buried stent syndrome occurred after at least 3 months, requiring an SEMS insertion.

Data regarding 30-day mortality and overall mortality are available for 106 patients. The 30-day mortality was 19.8% (21/106), whereas the overall mortality rate during follow-up was 36.8% (39/106). Most of the patients died due to underlying diseases, namely advanced malignancies, heart failure, renal and liver impairments.

### 3.5. Risk Factor Analysis

We performed univariable and multivariable regression analysis to assess factors independently associated with technical success, clinical success, AEs, and 30-day and overall mortality (Table 3 and Table 4).

A lower WBC count and stent diameter <10 mm correlated to technical failure in univariable analysis, but not in multivariable, while the choice of access (trans-gastric or trans-duodenal) was not associated with the technical outcome.

AC alone, namely cases not associated with other diseases or other complications, with a higher mean width of the gallbladder, 10 mm or wider stents, a shorter procedural time, early enteral feeding (within 48 h) and the absence of AEs, was significantly correlated to clinical success in univariable analysis. No correlation was shown with the route of drainage (trans-gastric or trans-duodenal). Multivariable analysis confirmed that AC alone (OR = 10.156, CI-95% 2.118–48.698; *p* = 0.004) and early enteral feeding (OR = 13.691, CI-95% 3.254–57.601; *p* < 0.001) are independently associated with clinical success.

AE occurrence was inversely correlated with intrachannel release of the second flange and early enteral feeding in univariable analysis, but these correlations were not confirmed in multivariable analysis. Again, no correlations were found with the route of drainage.

Moreover, in univariable analysis, AC complicated by or associated with other diseases, delayed enteral feeding (>48 h), together with higher levels of total and direct bilirubin, were predictors both for 30-day and overall mortality. On the other hand, 30-day mortality was inversely correlated with age, while overall mortality had analogous correlation with CRP and had a direct correlation with longer hospital stay and acalculous AC. In multivariable analysis, only complicated AC or AC associated with other diseases (OR = 41.7, CI-95% 1.979–879.0; *p* = 0.016) was a predictor of 30-day mortality, while delayed enteral feeding (OR = 8.582, CI-95% 1.698–43.381; *p* = 0.009), lower CRP levels before drainage (OR = 0.918, CI-95% 0.855–0.987; *p* = 0.020) and acalculous gallbladder disease (OR = 5.273, CI-95% 1.229–22.613; *p* = 0.025) were predictors of overall mortality.

### 3.6. Patients with Long-Term Survival

A subgroup analysis was performed among patients with a follow-up longer than one year (Table 5). A total of 40 (40/116, 34.5%) patients had a follow-up longer than one year, with a mean of 692 days (range 360–2090).

In this subset of patients, technical and clinical success were confirmed as close to optimal, at 95% (38/40) and 92.5% (37/40) respectively. A total of five patients experienced AEs (5/40, 12.5%); in three cases, AEs were intraprocedural, namely one bleeding, one LAMS malpositioning and one perforation, while the remaining two cases occurred during long term follow-up, at 27 and 516 days, and were represented by one buried stent syndrome and one stent migration, respectively.

Only one patient (1/40, 2.5%) had a recurrence of AC, which occurred 14 days after EUS-GBD and was managed using antibiotics; however, at long-term follow-up, particularly after 1 year, no recurrences of AC were reported.

A total of 13 patients (13/40, 32.5%) were admitted at least once during the overall follow-up, mainly due to an underlying disease (e.g., heart failure, chronic obstructive pulmonary disease, neurological diseases or neoplastic diseases). Only one patient was admitted because of an episode of biliary acute pancreatitis with the need for an ERCP.

None of the patients underwent surgical cholecystectomy or LAMS removal during the entire follow-up.

Long-term survival was good, with only five deaths (5/40, 12.5%) after 1 year (after a mean of 552 days) from the endoscopic procedure, and these were because of underlying diseases or other acute diseases (such as SARS-CoV-2 infection or renal failure).

In univariable analysis, no variables emerged as being statistically significant in relation to technical success, clinical success, adverse events or mortality. However, a trend toward significance emerged for the relationship between clinical and technical success and the absence of adverse events, and between high bilirubin levels after 2 weeks from EUS-GBD and mortality.

## 4. Discussion

The present study demonstrates that EUS-GBD using LAMS for AC is a valuable option for treatment of fragile patients showing high technical and clinical success rates (94% and 87.1%, respectively), with acceptable complications (10.3%). Most of the AEs occurred during the endoscopic procedure, and their severity varied from mild to severe (two mild, three moderate and seven severe) with no evidence of fatal ones. However, when considering overall mortality and 30-day mortality, rates were significantly high, up to 36.8% and 19.8%, respectively.

In univariable analysis, technical success was less likely for patients with a lower WBC count and drained with an LAMS < 10 mm in diameter, though these results were not confirmed in multivariable analysis. Shorter stents (6 × 8 mm and 8 × 8 mm) have been designed for EUS-guided biliary drainages, namely choledoco-duodenostomy (EUS-CDS), in which the space required for deployment is usually 15–20 mm [26]. In the case of EUS-GBD, usually, wider and longer stents are used (≥10 mm), because the diameter of the gallbladder in the case of AC is at least 40 mm, allowing for the opening of larger LAMS. In contrast to shorter stents, which require more control and precision, a wider diameter and longer saddle likely facilitate LAMS release, particularly in less experienced hands. Therefore, in this setting, a >10 mm LAMS should be preferred over smaller ones.

More controversial is the correlation between WBC and technical success: one hypothesis is that lower WBC may be the expression of AC which is being resolved, and, therefore, a lesser gallbladder distension is present that make LAMS deployment more challenging. In these cases, indeed, the space for the correct opening of the first flange could be insufficient, with an higher risk of stent misdeployment. Preliminary distension of the gallbladder with saline solution or a contrast injection through a 19 G needle and wire-guided stent deployment could be useful to reduce this risk.

These findings, however, unearth how a patient’s selection and the appropriate stent for the right indication are essential in order to increase the likelihood of a successful procedure.

Moreover, both univariable and multivariable analysis showed that early enteral feeding (within 48 h) had a positive correlation with clinical success and an inverse correlation with overall mortality; AC alone was a predictor of clinical success, whereas complicated AC or an association with other diseases was a predictor of 30-day mortality. As most cases of the “not alone” AC were associated with malignant diseases, it is clear that the clinical outcome in this setting is significantly worse, with an higher rate of 30-day mortality. Not least, multivariable analysis demonstrated that lower levels of CRP and acalculous gallbladder disease negatively influence overall mortality. This probably reflects the fact that an indication different from AC due to gallstones had worse outcomes when compared to other etiologies of AC. Indeed, the presence of acalculous AC and lower levels of CRP may reflect the presence of other severe underlying comorbidities, such as hepatic impairment and neoplastic diseases.

Interestingly, in our study, the endoscopist’s experience was not associated with worse outcomes or with higher rates of complications; similarly, the access of EUS-GBD, namely trans-gastric and trans-duodenal, was not associated with technical and clinical outcomes, nor adverse events. This slightly differed from European guideline’s recommendations on therapeutic EUS, which suggest the trans-duodenal route in order to reduce the risk of stent dysfunction [27].

Another point of note observed in this multicenter study is the long-term outcome. In at least 35% of our cohort, the follow-up was longer than 1 year (mean of 692 days), and, in subgroup analysis, outcomes were comparable to the overall follow-ups. Interestingly, when considering the rate of recurrence of AC, only one patient experienced a recurrent episode, which occurred more than 14 days after LAMS placement and was managed conservatively. Instead, after one year, no recurrence of AC was reported, even with 32.5% of this subset of patients being admitted at least once due to unrelated diseases. It is also notable that none of these patients underwent LAMS removal, demonstrating long-lasting patency and a good safety profile for these stents. In multivariable analysis, no factors emerged as predictors of long-term clinical outcomes, although a trend of significance was observed for mortality and the absence of AEs.

Previous studies reported comparable results in terms of technical and clinical success and adverse events [9,17,28], but most of the available data come from tertiary referral centers with skilled endoscopists. In our study, data were collected from several Italian centers, including both high-volume and secondary level centers with limited experience both in the use of LAMS and in EUS-GBD. Indeed, procedures were performed by endoscopists with different levels of experience in interventional endoscopy and in the use of LAMS. Data regarding the LAMS-training of the endoscopists included in the i-EUS group were recently reported, showing that 38.8% attended a dedicated training course, 27.7% were supported by an expert, 22.2% had both opportunities, and 8.3% had none [18]. These data were found to be reproducible and to reflect real-life settings. While previous studies showed that a learning curve correlates with longer procedural times and higher rates of AEs [17,28], in our study, endoscopist experience did not significantly affect the outcomes, showing that EUS-GBD provided satisfying results even when performed by less expert hands and still had a good safety profile. However, according to a recent meta regression analysis, despite the high rates of technical and clinical success, center experience proxied to >10 procedures/years increased and optimized the technical success and the overall clinical success [29].

Additionally, in our analysis, no fatal adverse events were reported, though a recent meta-analysis considering complications in EUS-GBD using LAMS [30] showed the rate of death was 5%. It is important to highlight that while the majority of the included studies reported death as the final outcome, most deaths were related to underlying diseases and none to the procedure. One limitation of most published studies is the lack of a classification of AEs according to a standardized severity index (e.g., ASGE), which would allow for a deeper comprehension of complications that affect this procedure and their management.

Despite the high clinical success rate and the absence of fatal AEs, the overall mortality and 30-day mortality rates were significantly higher in our cohort of patients than previously reported [17,31]. This could be related to the mean age of our study population, which was close to 83 years—at least 10 years higher than other studies [17,30]—and to the fact that at least 13% of the patients were affected by complications or associations with other diseases, which resulted as a negative predictor of 30-day mortality both at univariable and multivariable analysis. This is in line with previous evidence that suggests EUS-GBD outcome appears to be more influenced by patient conditions and underlying comorbidities rather than procedural success [31]. Indeed, the occurrence of AC probably indicates a worsening and exacerbation of the patient’s underlying comorbidities, which may have intervened later in the patient’s history, influencing long-term results.

Ours is the first study that demonstrates the role of enteral feeding in this setting and its association with clinical outcomes. Supposed mechanisms for this association may be different. First, patients with a better performance status, in which technical success was achieved and no adverse events occurred, intuitively received an early enteral feeding; in this case, the correlation with clinical success and 30-day mortality would be a consequence and not a cause of the outcome. Second, as is the case for acute pancreatitis and any other critical illness, inflammation is associated with intestinal failure with subsequent increased gut permeability, dysregulation of the immune response and dysbiosis [32,33,34]. Studies showed that when enteral feeding started within 24–48 h, thanks to reduced intestinal inflammation, there was a reduced risk of infection, multiorgan failure (MOF) and a reduced hospital stay [32,35,36]. This evidence could suggest that early enteral feeding should be mandatory to optimize patient outcomes.

To the best of our knowledge, this is the largest cohort of patients with a >1-year follow-up that shows that this technique has satisfactory long-term outcomes, with a near-optimal safety profile. Previous studies had a follow-up of at least one year [17,31], but long-term outcomes were not evaluated selectively, apart from overall mortality.

Interesting results on long-term outcomes for the treatment of AC have recently been published on ETGBD using double-pigtail plastic stents [37]. According to a study on 49 patients, clinical success was achieved in all cases and demonstrated long-term efficacy in 96% of patients. A higher risk of recurrence of AC was shown when a single stent was used, while the placement of two was a protective factor against the need for elective repeated ETGBD. However, the cannulation of the cystic duct, and, above all, its placement, could be challenging and not always achievable. Although EUS-GBD showed better technical and clinical success rates [38,39,40], a more aggressive ETGBD using two double-pigtail plastic stents could represent a valuable alternative with satisfactory long-term outcomes [41]. A head-to-head study comparing EUS-GBD using LAMS and ETGBD using two double-pigtail plastic stents would be needed to evaluate whether one technique is superior to the other.

The present study also has several limitations, such as the retrospective design, which may affect several reports (such as complications), the lack of information about patient comorbidities and the involvement of many centers that are inhomogeneous in terms of volume and experience. Moreover, due to the scarcity of significant variables in the univariable model, and the low number of events (technical failure), we did not perform a multivariable analysis that would have allowed us to establish an independent relationship between stent size and technical failure, and control, at the same time, of other possible confounders.

Nonetheless, we believe that this study provides a truthful picture of the real-life setting, which should be taken into account when considering EUS-GBD as an option for treatment.

## 5. Conclusions

This multicenter study shows how, in a real-life setting, including both secondary and tertiary referral Centers, EUS-GBD using LAMS has both high technical and clinical success rates, with a non-negligible rate of AE, though none were fatal. In a follow-up longer than one year, no recurrence of AC was observed, demonstrating satisfactory patency of these devices, with a good safety profile even when left in place. Therefore, according to our data, EUS-GBD using LAMS could represent a valuable, long-lasting option for treatment of fragile patients.

## Figures and Tables

**Table 1:** Patients and clinical characteristics.

Variables	*n* (%) or Mean ± SD
female	56/116 (48.3%)
age, years	82.7 ± 11
diagnosis	
- acute cholecystitis alone	101/116 (87.1%)
- malignant obstruction	10/116 (8.6%)
- empyema	3/116 (2.5%)
- Mirizzi’s syndrome	1/116 (0.8%)
- cholecysto-duodenal fistula	1/116 (0.8%)
warfarin or DOAC intake	14/116 (12%)
previous ERCP indication	
- stone extraction	28/116 (24.3%)
- transpapillary gallbladder drainage	3/116 (2.6%)
concomitant percutaneous drainage	3/116 (2.6%)
gallbladder features	
- stone and sludge	63/116 (54.3)
- acalcolous	14/116 (12.1)
- purulent	39/116 (33.6)
gallbladder length, mm	71.2 ± 35.8
gallbladder width, mm	59.4 ± 34
WBC Count (×10^3^) *	16.13 ± 6.06
Total Bilirubin Levels (mg/dL) *	5.15 ± 6.32
Direct Bilirubin Levels (mg/dL) *	4.14 ± 5.15
CRP (mg/dL) *	21.21 ± 17.5

* Mean values at baseline.

**Table 2:** Adverse events and management.

N°	Type	Severity	Timing	Management	Other
1	bleeding	mild	intraprocedural	conservative	
2	bleeding	mild	intraprocedural	endoscopy	hemostasis
3	AC recurrence	moderate	early	conservative	
4	dislodgement	moderate	intraprocedural	endoscopy	placement of OTSC
5	dislodgement	moderate	intraprocedural	endoscopy	placement of second LAMS
6	dislodgement	severe	intraprocedural	surgery	urgent cholecystectomy
7	migration	severe	delayed	surgery	open cholecystectomy +suturing
8	malpositioning	severe	intraprocedural	endoscopy	LAMS positioned in renal cyst. Removal of LAMS
9	perforation	severe	delayed	surgery	open cholecystectomy + suturing
10	perforation	severe	intraprocedural	surgery	VLS cholecystectomy + suturing
11	buried stent	severe	delayed	endoscopy	SEMS insertion
12	cardiac arrest	severe	intraprocedural	conservative	

Abbreviations. OTSC: over-the-scope clip; LAMS: lumen-apposing metal stent; SEMS: self-expandable metal stent; AC: acute cholecystitis; VLS: videolaparoscopic.

**Table 3:** Univariable and multivariable analysis of variables associated with technical success, clinical success and adverse events.

Technical Success					
	Univariable Analysis			Multivariable Analysis	
Variable	Technical Success (*n* = 109)	Technical Failure (*n* = 7)	*p* Value	Odds Ratios (CI-95%)	*p* Value
WBC (mean ± SD)	18,207 ± 15,076	11,500 ± 3316	0.020	-	
stent size (width)			0.002	-	
- <10 mm	3 (3.1)	2 (28.6)			
- ≥10 mm	95 (96.9)	5 (71.4)			
**Clinical Success**					
	**Univariable Analysis**			**Multivariable Analysis**	
**Variable**	**Clinical Success** **(*n* = 101)**	**Clinical Failure** **(*n* = 15)**	***p* Value**	**Odds Ratios (CI-95%)**	***p* Value**
diagnosis			0.019	10.156 (2.118–48.698)	0.004
- acute cholecystitis	90 (89.1)	10 (66.7)			
- other	11 (10.9)	5 (33.3)			
gallbladder width, mm (mean ± SD)	60.9 ± 36.5	42.1 ± 25.4	0.036	-	
stent size (width)			0.053	-	
- <10 mm	3 (3.2)	2 (15.4)			
- ≥10 mm	90 (96.8)	11 (84.6)			
procedure time, minutes (mean ± SD)	22.9 ± 16.5	34.5 ± 24.4	0.020	-	
beginning of post-procedural enteral diet				13.691 (3.254–57.601)	<0.001
- immediate or within 48 h	86 (86.0)	7 (46.7)	<0.001		
- after 48 h	14 (14.0)	8 (63.3)	0.003		
adverse events	6 (5.9)	6 (60.0)	<0.001	-	
**Adverse Events**					
	**Univariable Analysis**			**Multivariable Analysis**	
**Variable**	**Adverse Events** **(*n* = 12)**	**no Adverse Events** **(*n* = 104)**	***p* Value**	**Odds Ratios (CI-95%)**	***p* Value**
release of the 2nd flange			0.046	-	
- intrachannel	5 (41.7)	73 (70.2)			
- endoscopic view	7 (58.3)	31 (29.8)			
beginning of post-procedural enteral diet			0.036	-	
- immediate or within 48 h	7 (58.3)	86 (83.5)			
- after 48 h	5 (41.7)	17 (16.5)			

Abbreviations. SD: standard deviation.

**Table 4:** Univariable and multivariable analysis of variables associated with 30-day mortality and overall mortality.

30-Days Mortality					
	Univariable Analysis			Multivariable Analysis	
Variable	30-Day Death (*n* = 21)	30-Day Survival (*n* = 85)	*p* Value	Odds Ratios (CI-95%)	*p* Value
age, years (mean ± SD)	78.1 ± 14.5	83.7 ± 9.9	0.038	1.031 (0.946–1.122)	0.488
total bilirubin, mg/dL (mean ± SD)	10.9 ± 7.6	4.4 ± 5.8	0.047	1.012 (0.872–1.173)	0.879
diagnosis			<0.001	41.7 (1.979–879.0)	0.016
- acute cholecystitis	13 (61.9)	77 (90.6)			
- other	8 (38.1)	8 (9.4)			
beginning of post-procedural enteral diet			0.005	8.578 (0.545–134.197)	0.126
- immediate or within 48 h	14 (66.7)	77 (90.6)			
- after 48 h	7 (33.3)	8 (9.4)			
**Overall Mortality**					
	**Univariable analysis**			**Multivariable analysis**	
**Variable**	**Overall Mortality** **(*n* = 39)**	**Overall Survival** **(*n* = 67)**	***p* Value**	**Odds Ratios (CI-95%)**	***p* Value**
CRP, mg/dL (mean ± SD)	12.2 ± 8.7	22.9 ± 18.4	0.005	0.918 (0.855–0.987)	0.020
total bilirubin, mg/dL (mean ± SD)	9.1 ± 8.7	2.9 ± 2.7	<0.001	1.058 (0.960–1.166)	0.259
diagnosis			0.001	7.542 (0.972–58.510)	0.053
- acute cholecystitis	27 (69.2)	63 (94.0)			
- other	12 (30.8)	4 (6.0)			
beginning of post-procedural enteral diet			0.002	8.582 (1.698–43.381)	0.009
- immediate or within 48 h	28 (71.8)	63 (94.0)			
- after 48 h	11 (28.2)	4 (6.0)			
length of hospitalization post procedure, days (mean ± SD)	13.9 ± 11.7	9.6 ± 7.4	0.021	0.954 (0.899–1.013)	0.125
gallbladder disease			0.007		
- stone and sludge (ref)	14 (36.8)	40 (59.7)			
- acalcolous	10 (26.3)	4 (6.0)		0.306 (0.026–3.541)	0.343
- purulent	14 (36.8)	23 (34.3)		5.273 (1.229–22.613)	0.025

Abbreviations. SD: standard deviation.

**Table 5:** Long-term outcomes.

Variable	*n* (%) or Median + Range
patient number	40 /116 (34.5)
follow-up, days	692 (360–2090)
technical success	38/40 (95)
clinical success	37/40 (92.5)
adverse events	5/40 (12.5)
- - intraprocedural	3/5 (60)
- - during follow up	2/5 (40)
acute cholecystitis recurrence	1/40 (2.5)
surgical cholecystectomy	0/40 (0)
mortality	5/40 (12.5)

## Data Availability

Data is contained within the article.

## Supplementary Materials

The following supporting information can be downloaded at: https://www.mdpi.com/article/10.3390/diagnostics14040413/s1, Supplementary Table S1: Type of LAMS used.
