# Supplementary material for: EUS-Guided Gallbladder Drainage Using a Lumen-Apposing Metal Stent for Acute Cholecystitis: Results of a Nationwide Study with Long-Term Follow-Up
Source: Diagnostics (Basel). 2024 Feb 13;14(4):413. doi: 10.3390/diagnostics14040413 (PMC10887962; doi:10.3390/diagnostics14040413)
Supplement: Supplementary file 1 [file diagnostics-14-00413-s001.zip › diagnostics-2770498-supplementary.pdf]

Supplementary Table S1. Type of LAMS used and technique.

Variable	N (%)
Stent type	
• Hot axios	107/116 (92.2)
• Nagi	9/116 (7.8)
Hot axios Stent dimension (width x length)	
• 10 mm x 10 mm	82/116 (70.7)
• 15 mm x 10 mm	19/116 (16.4)
• 8 mm x 8 mm	4/116 (3.4)
• 6 mm x 8 mm	1/116 (0.9)
• 20 mm x 10 mm	1/116 (0.9)
Nagi Stent dimension (width x length)	
• 12 mm x 20 mm	7/116 (6.3)
• 16 mm x 20 mm	1/116 (0.9)
• 12 mm x 30 mm	1/116 (0.9)
Gallbladder access	
• Transgastric	51/116 (44.8)
• Transduodenal	62/116 (53.3)
• Transjejunal	2/116 (1.7)
Step for access into common bile duct	
• single stage	101/116 (87.1)
• multiple steps	15/116 (12.9)
Release of the 2nd flange	
• Intrachannel	76/116 (67.2)
• Under fluoroscopic view	45/116 (38.8)
